# Microbial composition of archaeological middens: tracing human footprints through centuries in Greenland’s ancient settlements

**DOI:** 10.3389/fmicb.2026.1809037

**Published:** 2026-06-17

**Authors:** Lorrie Maccario, Saria Otani, Judit Szarvas, Louise Hindborg Mortensen, Bo Elberling, Kirstine Eiby Møller, Christian Edelfelt Koch Madsen, Frank M. Aarestrup, Anders Priemé

**Affiliations:** 1 Department of Biology, University of Copenhagen, Copenhagen, Denmark; 2 National Food Institute, Denmark Technical University, Kongens Lyngby, Denmark; 3 Department of Geosciences and Natural Resource Management, University of Copenhagen, Copenhagen, Denmark; 4 Greenland National Museum & Archives, Nuuk, Greenland

**Keywords:** archaeological microbiomes, climate change, middens, Norse settlements, soil microbiomes

## Abstract

The history of Greenland is marked by different waves of Paleo-Inuit immigration from North America from 2,500 BC to the 12th century and from the 10th to 15th century, Norse settlers immigrated from Northwest Europe and flourished in Southwest Greenland with the introduction of domestic livestock. The different Inuit and Norse cultures created middens by dumping and accumulating domestic waste; a latent source of microbes, including potential pathogens, that might have been preserved due to the general wet and cold conditions in the region. The aim of this study was to evaluate whether ancient Arctic settlements might be possible hot-spots for pathogenic agents that may spread to the surrounding environment because of current climate changes. Using metagenomics, we compared the microbial communities and resistomes of 78 samples from middens from different ages and locations in West and South Greenland (two Paleo-Inuit, four Norse and one early Colonial-time middens) to 143 soil samples from nearby surroundings. We found that the middens harbor a distinctive microbial signature enriched in human-associated bacteria. Those include opportunistic pathogens such as *Clostridium perfringens* and *Paeniclostridium sordellii*. In some early colonial midden layers, *C. perfringens* and *Paraclostridium tenue* together accounted for up to ~40%–50% of MetaPhlAn-derived relative abundance in individual samples. Antimicrobial resistance genes representing 17 resistance classes were detected across all sites, dominated by *β*-lactam and tetracycline resistance. Transect analyses across an actively eroding midden showed that midden-derived bacteria were confined to local erosion layers and were rapidly replaced by native marine communities, indicating limited environmental dispersal.

## 1 Introduction

The history of Greenland is marked by different waves of Paleo-Inuit immigration from North America from 2,500 BC to the 12th century. From the 10th to 15th century, Norse settlers immigrated from Europe and flourished in southwest Greenland with the introduction of domestic livestock (Madsen, 2014). The different Inuit and Norse cultures created middens by dumping and accumulating domestic waste. Today, animal bones, excrements, mollusc shells and other artefacts associated with past human occupation are a valuable resource for archaeologists to study these past societies’ diets, habits, life and death (Madsen, 2014). However, these archaeological features might also represent unique microbial reservoirs of organisms from mixed origins (soil-, human- or animal-associated) within an organic rich matrix; a latent source of microbes, including potential pathogens. Importantly, some of these might have been preserved due to low temperatures or permanently frozen soil near water-saturated conditions. However, currently the soil is exposed to warming, changes in precipitation, increased drainage and a potential collapse and erosion. Arctic temperatures are rising three-four times faster than the global average (Rantanen et al., 2022) leading to widespread permafrost thaw and the destabilization of long-frozen landscapes (Abbott and Jones, 2015; Biskaborn et al., 2019). As the protective permafrost layer thaws, midden material may be carried away as permafrost erodes (Abbott and Jones, 2015) or thaws gradually with increasing active layer depth (Koven et al., 2011). Permafrost erosion happens along streams, rivers, coastlines, and lake shores where it leads to abrupt displacement of permafrost material, including microorganisms, to non-frozen aquatic environments (Vonk and Gustafsson, 2013). Gradual thawing of permafrost through a deepening of the active layer does not lead to displacement of bulk permafrost material but may result in enhanced translocation of water, solutes and microorganisms when permafrost ice is transformed into water (Zastruzny et al., 2024). Rising sea levels and loss of sea ice (Nielsen et al., 2022) also accelerates the erosion of middens that are often situated along the coast and midden material might be washed out into the sea. This raises the question whether these sites represent a disease emergence threat.

An example of possible release is the 2016 outbreak of Anthrax believed to have emerged from *Bacillus anthracis* spores from thawing permafrost in the Yamal Peninsula in Russia, which killed over 2,000 reindeer and caused at least one human death (Ezhova et al., 2021). In addition to this, viable viruses capable of infecting amoeba were recovered from permafrost in Siberia (Alempic et al., 2023). Knowledge about microbial DNA signatures preserved in archaeological settlements would allow a more detailed analysis of the emergence and evolution of these organisms because of climate change, enabling improved risk assessments.

Importantly, the current and impending climate changes are not only increasing the exposure and release from the ancient deposit exposures (Fenger-Nielsen et al., 2020) but are also having a profound impact on both local and migrating bird and mammal populations, as well as human populations, including plans for increasing industries, trade and human visitation and local anthropogenic activity. Consequently, changing the interactions between humans and the Arctic ecosystem and increasing risk contact with midden material or animal vectors. Thus, a shift to increased transarctic migration, as well as a shift from a migratory strategy to high-arctic year-round residency has been predicted (Clairbaux et al., 2019). For influenza virus it has been shown how fast migrating birds can spread viruses across the globe, with opportunity for spread through layovers of migratory birds in Iceland and Greenland (Günther et al., 2022).

Domestic livestock - cattle, goats, sheep, chickens, dogs, and cats - was reintroduced with the Danish-Norwegian colonization of Greenland from 1721. From the late 1800s, livestock farming with cattle and sheep was increasingly taken up in South and Southwest Greenland and has continued to this day. With ongoing climate changes the increase in plant production in West Greenland is opening for the development of sheep farming in the Nuuk area, exactly where sheep farms were situated during the Norse era and abandoned for the past 600 years (Westergaard-Nielsen et al., 2015).

The aim of this study was to evaluate if ancient Arctic settlements might be possible hot-spots for pathogenic agents affecting humans or livestock, and if potential pathogens in the future may spread to the surrounding environment. Using metagenomics, we compared the microbial communities of middens from different ages and locations in West and South Greenland (Paleo-Inuit, Norse and early colonial-time middens) and compared them to surrounding soils.

## 2 Methods

### 2.1 Sample collection

**Figure 1:**
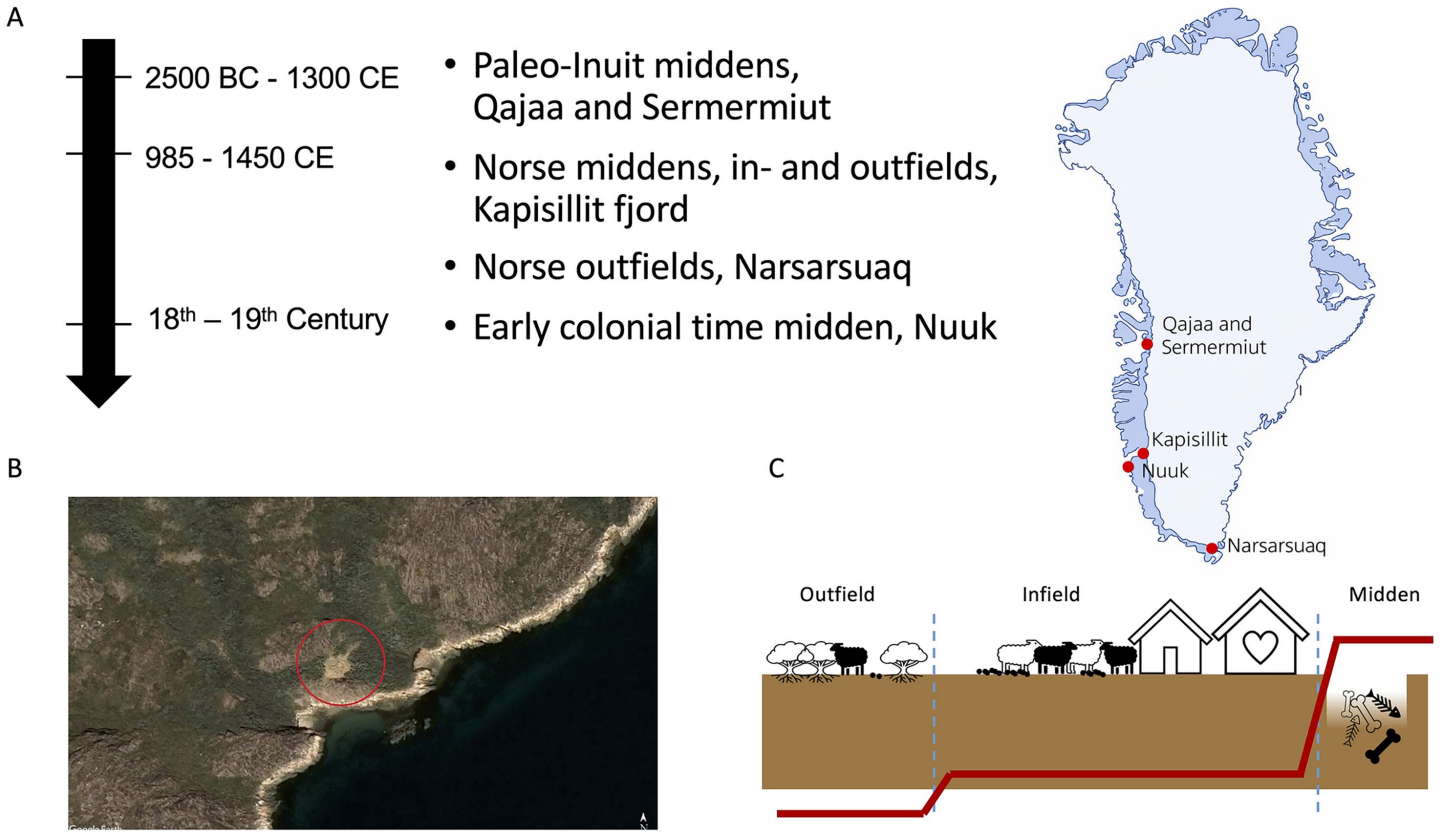
Sampling of middens for microbial community characterization: **(A)** Sampling locations and approximative age. **(B)** Satellite picture of a Norse midden site along the coast of Kapissilit fjord. **(C)** Schematic representation of Norse settlements with outfields used for summer grazing, infields used as winter enclosures for livestock, and middens.

In total, 221 soil and midden samples (143 from soil and 78 from middens) were collected from Greenland over two sampling campaigns (2018 for Qajaa and September 2020 for the other sites) (Supplementary Table S1). The 78 midden samples were from three historical populations/cultures: including Paleo-Inuit (2,400 BC–1200 AD) (nine from Qajaa, 12 from Sermermiut), Norse (1,000 AD–1500 AD) (34 from Kapisilit), and early colonial-time Modern Inuit (1800 AD) (23 from Nuuk) (Figure 1). The midden sites were identified using the Greenland National Museum and Archives registry. The Paleo-Inuit middens are situated at Qajaa and Sermermiut in the Ilulissat Icefjord in the western central part of Greenland. Further details about the Qajaa site can be found in Elberling et al. (2011). The Norse middens are located along the coast of the Kapissilit Fjord ca. 80 km ENE of Nuuk, while the Modern Inuit midden from the early colonial era is located at the Noorliit site in Nuuk. At the Norse settlement at Kapisilit, we also collected 50 samples from so-called infields (winter enclosures for livestock at the settlements) and 30 outfields (summer grazing grounds for livestock) (Supplementary Table S1). In addition, 48 soil samples (out-field) were collected 5 km from the Norse settlements near Narsarsuaq, Southern Greenland (Supplementary Table S1).

Sample collection procedure: the samples from the Qajaa midden were collected by drilling to different permafrost depths using a 4.5-cm corer. The middens sampled during the 2020-field campaign were excavated by digging a pit until reaching the permafrost layer, and the presence of midden material was confirmed through the identification of archaeological artifacts. Midden samples were collected from various midden layers or at 10 cm intervals if specific layers could not be identified. In addition, we collected samples from specific archaeological artifacts that were identified. The soil was manually homogenized and approximately 1 g of subsample stored in vials with a preservative (LifeGuard Soil Preservation Solution, Qiagen) until DNA extraction. Samples of surrounding outfield soils were collected from 0 to 5 cm and 5 to 10 cm layers, homogenized, and stored in the same manner.

### 2.2 DNA purification and sequencing

Total DNA was extracted from all samples using the QIAamp Fast DNA Stool Mini Kit (Qiagen, Germany) according to the manufacturer’s protocol, with 200 mg of material as input. DNA was eluted in 50 μL of pre-heated (65 °C) AE buffer to maximize yield and quantified using a Qubit Fluorometer (Thermo Fisher Scientific). DNA from all samples was fragmented to 300 bp using transposases during the metagenomic library preparation using the PCR-free KAPA HyperPrep Kit (Roche) and sequenced on an Illumina NovaSeq 6,000 S4 platform (2 × 150 bp). No ancient-DNA-specific damage repair, uracil-DNA glycosylase treatment, or post-mortem authentication procedures were performed. Because DNA extracts were fragmented during the library preparation, read-length distributions of the final sequencing libraries do not reflect the original fragment-length distribution of DNA molecules in the samples and were therefore not used as an authentication metric.

### 2.3 Metagenomic data analyses

Metagenomic data from the samples were analyzed for bacterial community composition and antimicrobial resistance genes (ARGs). All software was used with default parameters unless notified. Raw sequence reads were cleaned using FastP v0.23.2 (Chen et al., 2018) with paired-end reads merging (with unmerged options) and adapter auto-detection. Metaphlan4 v4.0.2 (mpa_vJan21_CHOCOPhlAnSGB_202103) (Blanco-Míguez et al., n.d.) was used for taxa assignment, it is a clade-specific marker-based taxonomic profiler to estimate relative taxonomic abundances from reads mapping to curated marker genes. To quantify the fraction of each metagenome represented in the MetaPhlAn reference space, we calculated the estimated MetaPhlAn marker-covered fraction from the merged MetaPhlAn profiles as 100 − UNCLASSIFIED. We summarized this value by sample group and reported per-sample values in Supplementary Table S3. We also summarized observed MetaPhlAn richness at species, genus, and family level. ARGs were assigned using KMA and the ResFinder database (10-2021) (Bortolaia et al., 2020; Clausen et al., 2025). ARGs were called if the read had 120 bp minimum coverage with at least two fragments assigned to it. The hits were then aggregated at 90% homology, and assigned to class levels as well. Statistical analyses were performed in R including the packages phyloseq (McMurdie and Holmes, 2013), ape (Paradis and Schliep, 2019), stringr (Wickham, 2022), picante (Kembel et al., 2010), ggplot2 (Wickham, 2016), DAtest (Russel et al., 2018), ggrepel (Slowikowski, 2026), dplyr (R Foundation for Statistical Computing, 2023), ggpubr (Wickham, 2023), MicEco (Russel, 2021), vegan (Oksanen et al., 2019), RColorBrewer (Neuwirth, 2022).

## 3 Results and discussion

### 3.1 Midden bacterial community taxonomic signature

In this study, we compared microbial community composition, both in terms of taxonomy and ARG occurrence, from three different types of middens (paleo-Inuit, Norse and early colonial Inuit) to outfield soils to investigate if we could observe a trace from these past archeological artifacts and detect potential pathogens. Because no dedicated aDNA authentication analyses were performed, including fragment-length or terminal damage profiling, the detected microbial DNA should be interpreted as preserved DNA signatures in archaeological deposits rather than definitive evidence of authenticated ancient or viable microorganisms.

Sequencing resulted in 69 to 238 M reads between the midden metagenomes. MetaPhlAn profiling identified 1,207 bacterial species across all midden samples, with 9 to 202 bacterial species detected per sample (median 33). However, because MetaPhlAn is a clade-specific marker-based profiler, these values reflect observed richness within the MetaPhlAn reference-covered fraction of the metagenomes rather than total community richness. Consistent with this, the estimated MetaPhlAn marker-covered fraction was low overall (Supplementary Table S3). Accordingly, the MetaPhlAn-based taxonomic results should be interpreted as comparative profiles of the reference-covered fraction of the communities rather than as an exhaustive census of total bacterial diversity.

**Figure 2:**
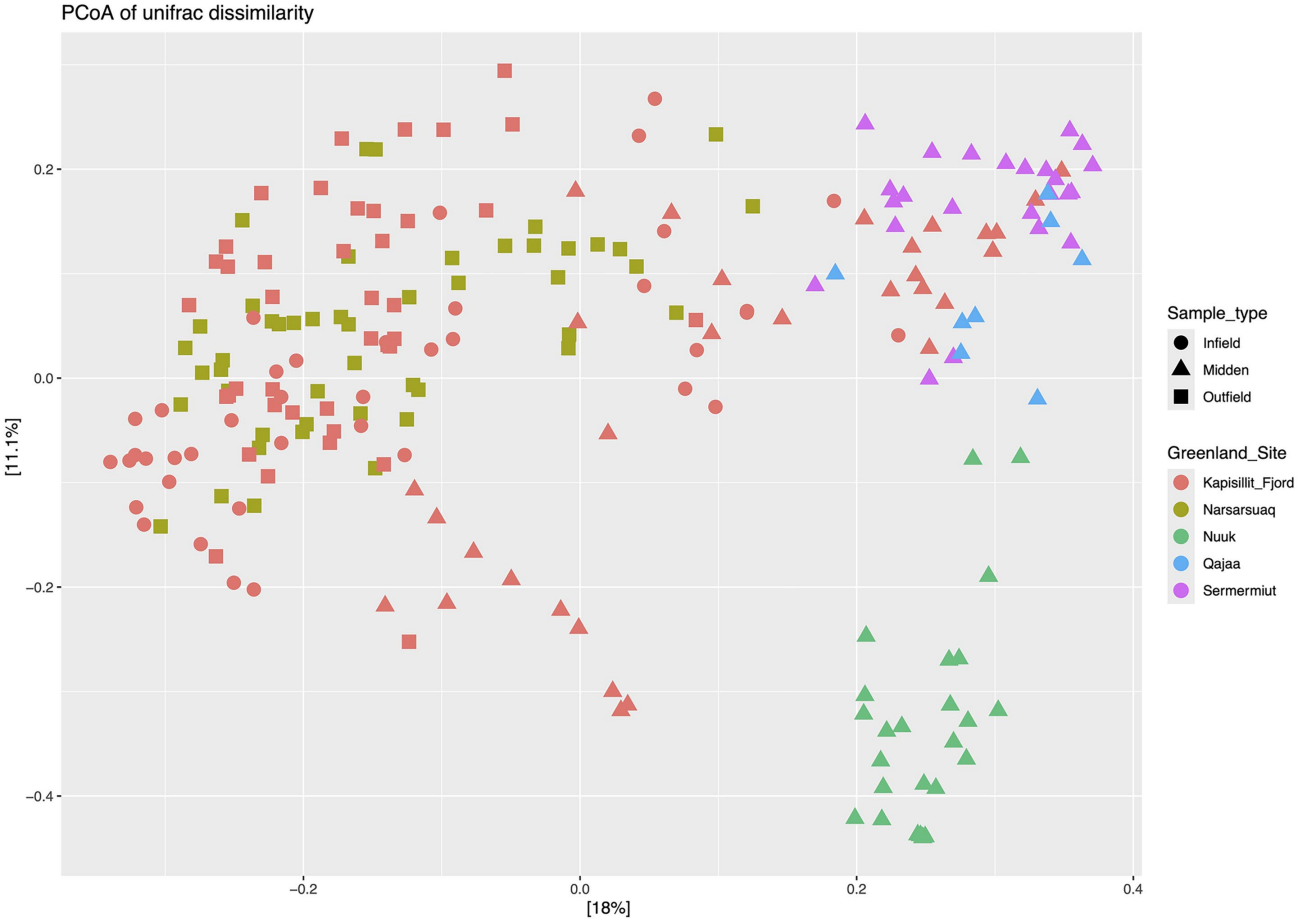
Dissimilarity in bacterial community composition: principal coordinates analysis (PCoA) using UniFrac distance and based on the occurrence of species genomic bins annotated with Metaphlan4 profiler in samples from the different Greenlandic sites Kapisillit, Nuuk, Qajaa, Sermermiut, and Narsarsuaq. Sample types are indicated (identified middens or surrounding soil from Norse infields or outfield). Permutational analysis of variance (Adonis test with 1,000 permutations) showed a significant effect of soil type, middens, infields, or outfields on shaping the microbial community composition (R^2^ = 14.9%, *p* < 0.001). Because MetaPhlAn captured only a limited reference-covered fraction of many samples, this ordination should be interpreted as an exploratory comparison of broad compositional differences within the MetaPhlAn-detectable fraction of the bacterial communities.

Beta diversity analyses using PCoA based on unweighted UniFrac distance (Figure 2) showed a significant impact of midden origin (*p*-value < 0.001, *R*^2^ = 0.1647) and location (*p*-value < 0.001, *R*^2^ = 0.24432) on the MetaPhlAn-detectable fraction of the bacterial community. Particularly, early colonial time Nuuk midden bacterial communities had the most distinct bacterial communities compared to the rest of the samples, while the dissimilarity became more diluted in the others samples, especially between the outfield and infield soils in Kapisillit and Narsarsuaq (Figure 2). However, since these are based on low detection rate of MetaPhlAn, we therefore interpret this ordination as an exploratory comparison of broad compositional differences between sample types rather than a complete representation of community turnover.

**Figure 3:**
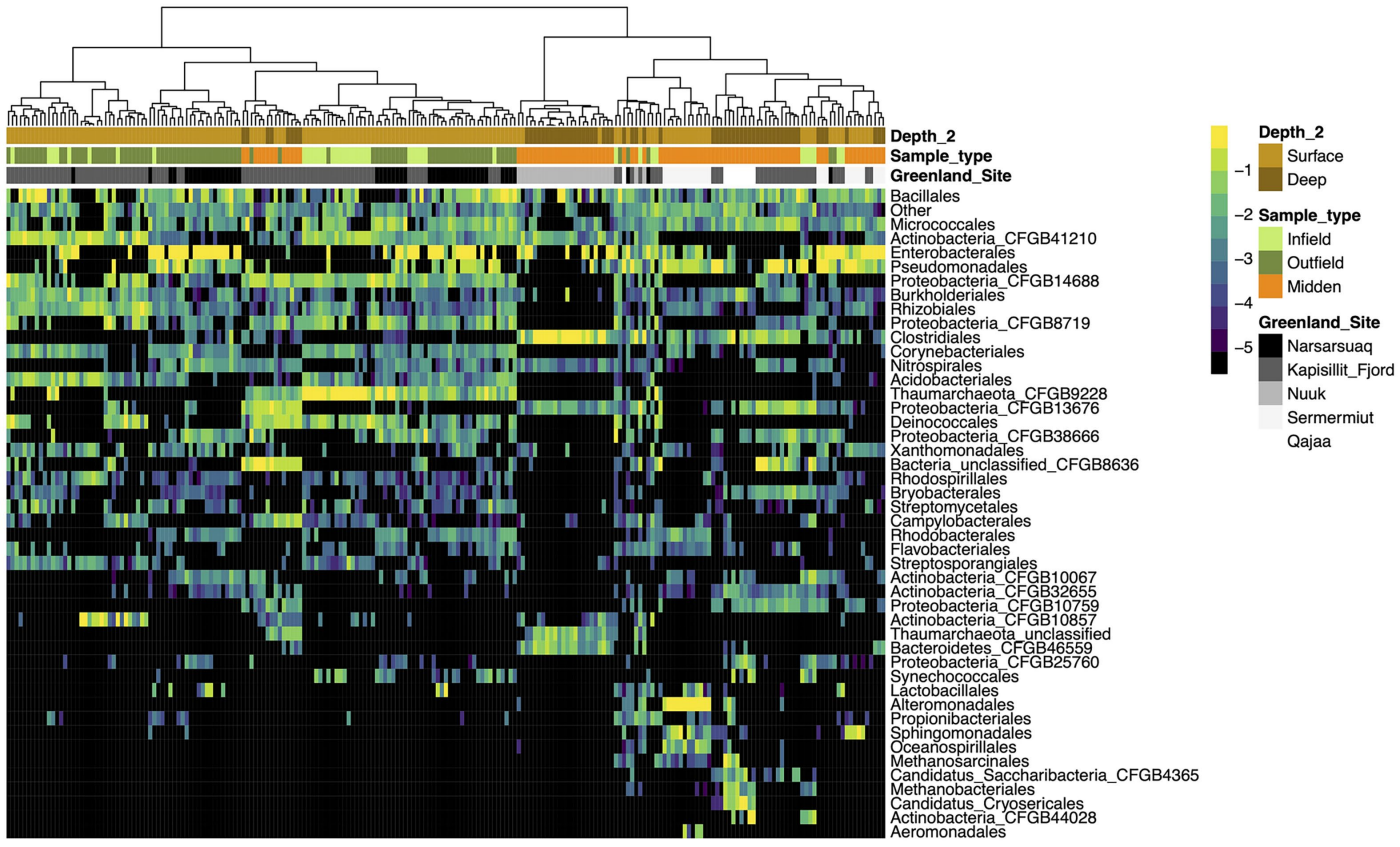
Microbial community structure: Heatmap of the relative abundance (Log_10_ transformed) of the 47 most abundant bacterial orders. Clustering was performed using UniFrac distance and ward. D2. All taxa with relative abundance lower than 0.1% were grouped as “Other”.

Based on the 47 most abundant bacterial orders (Figure 3), we observed two clear patterns. The samples clustered by environment type (midden material, infield, outfield, surface vs. deep), indicating that the bacterial communities are structured by site context rather than being homogeneous across the landscape. Many of the dominant lineages were only partially classified and remain annotated at high taxonomic ranks such as Proteobacteria, Actinobacteria, and Thaumarchaeota, or as unclassified families within these groups. This limited resolution highlights how poorly described Arctic soils and archaeological deposits remain. However, examples of dominant taxa in each group of samples were observed. The group of *Rhizobiales*, that are well studied soil plant-associated bacteria and contains many genera of nitrogen-fixing, methanotrophic, legume-nodulating, microsymbiotic bacteria (Garrity et al., 2004) were not detected in most middens samples, especially the Nuuk early colonial midden. On the contrary, *Clostridiales* were highly abundant in middens samples, especially again in the early colonial Nuuk midden (youngest waste). This group of bacteria can be isolated from numerous environments including soil and human intestines and also contains agents of some of the most devastating diseases.

To further address which bacterial taxa can be distinctive in the midden bacterial community composition, we performed a differential abundance analysis of all midden samples, independently of their geographic location, against all infield and outfield soils (Supplementary Figure S1). Several taxa that were more prominent in midden-associated MetaPhlAn profiles than in soil-associated profiles were host-associated and/or pathogenic bacteria (Bartlett et al., 2022). Among them, we detected commensal bacteria commonly found in human fecal matter such as *Clostridium massilliamazoniense* (Dione et al., 2020), agents of food poisoning such as *C. perfringens* (García et al., 2023), *C. baratii* that can cause rare infant or adult botulism (Tréhard et al., 2016) and *Paeniclostridium sordellii* causing pneumonia, endocarditis, arthritis, peritonitis, and myonecrosis and in rare cases severe toxic shock syndrome (Aronoff, 2013). The relative abundance of other non-spore forming Firmicutes were also increased in middens including *Ramboutsia hominins* and *R. timonensis* and *Paraclostridium tenue*. Both *Ramboutsia* spp. are anaerobes that were recently isolated from the human gut and have been detected as members of the human and animals gut microbiota (Biagi et al., 2020) but are rarely described from environmental samples. *P. tenue* was originally isolated from post-abortion abscess and has been listed as a human established pathogen (Bartlett et al., 2022).

We next analyzed the relative abundance of the midden-enriched taxa across the samples and in samples directly associated with visible archaeological artifacts (Supplementary Figures S2A,B). Most taxa occurred at markedly higher relative abundance in midden samples than in the surrounding pristine soils, confirming that these deposits preserve distinct, host-associated bacterial signatures. Among them, *C. perfringens* and *P. tenue* reached the highest levels, occasionally accounting for up to 40%–50% of the MetaPhlAn-derived relative abundance in individual samples, while *R. hominis*, *R. timonensis*, and *P. sordellii* were detected at lower but consistent abundance. These organisms are typical anaerobic members of animal and human intestinal microbiota and include opportunistic or toxin-producing species [e.g., *C. perfringens* (García et al., 2023)], suggesting persistence of gut- or carcass-associated bacteria in the middens.

The early colonial-era Nuuk midden, particularly layers containing decomposing seal skin, was dominated by *C. perfringens* and *P. tenue*, whereas Norse-period middens from Kapisillit associated with decomposing bones contained higher proportions of unclassified *Proteobacteria* and *Clostridiaceae* lineages (Supplementary Figure S2B). This pattern indicates that substrate type and midden age both shape the resident bacterial community: younger middens rich in organic domestic waste harbor more faecal and necrotic-tissue anaerobes compared to the older Norse middens while Paleo-Inuit deposits retain a more soil-like, environmental profile. These results support the view that Arctic middens can act as localized reservoirs of host-associated bacteria that can remain detectable for centuries, probably linked to burial conditions (degree of water saturation and low temperatures).

### 3.2 Local compositional turnover across an eroding midden gradient

**Figure 4:**
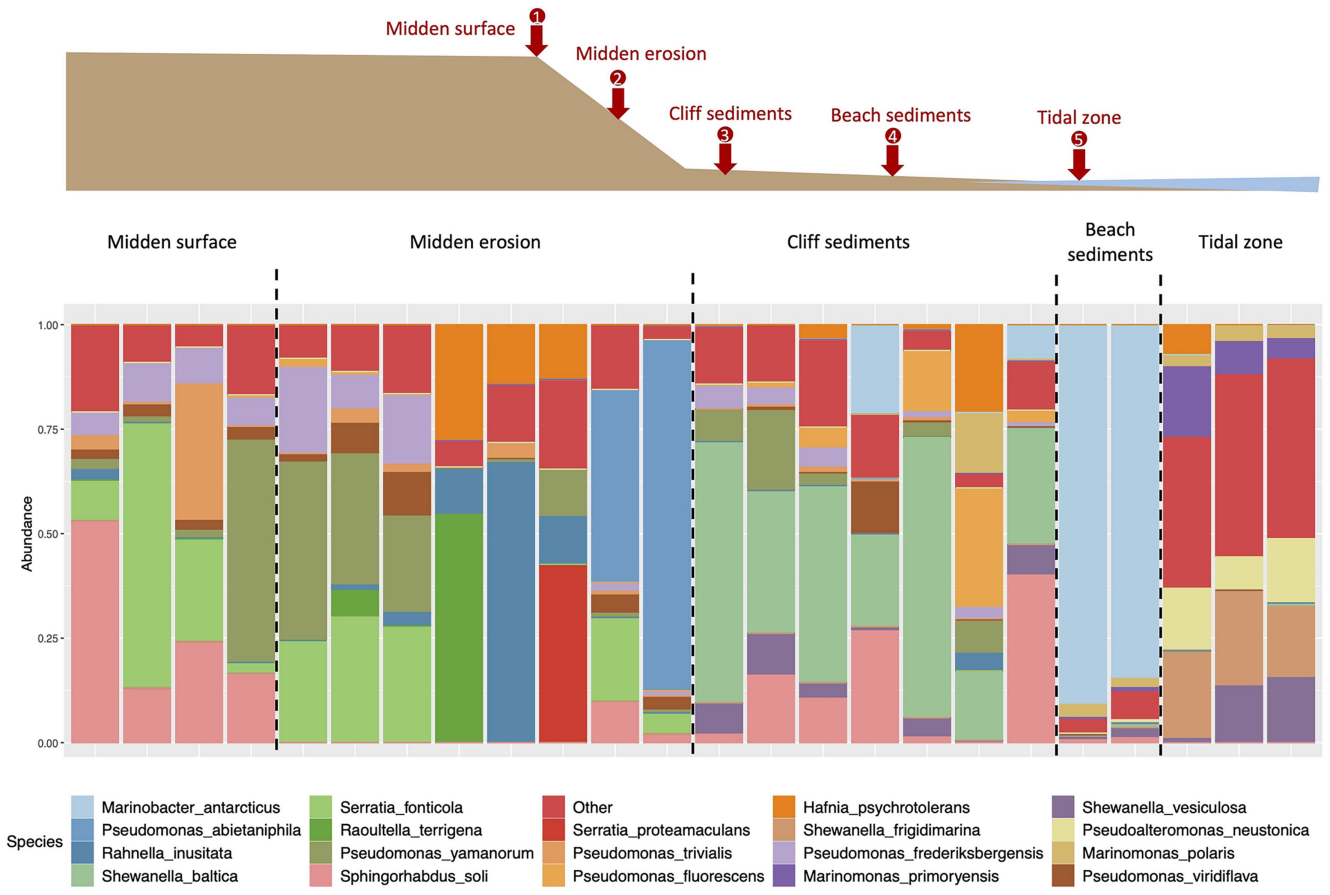
Bacterial community composition along the Paleo-Inuit midden erosion gradient at Sermermiut: Barplot of the relative abundance of the 20 most abundant species in midden surface, the erosion front, cliff sediments, beach sediments, and tidal zone.

To examine whether midden-associated bacterial signature could still be detected outside the exposed deposits, we investigated the Sermermiut site where the deposit is actively eroding into the coastal zone. Samples were collected along a transect from the midden surface through the eroding face, cliff sediments, beach sediments, and the tidal zone (Figure 4). We chose to assess the entire bacterial community compositions and not just potentially pathogenic taxa to increase sensitivity and detect compositional changes along the gradient. The bacterial community composition changed sharply along this gradient, with increasing distance to the midden and reflecting a transition from terrestrial to marine ecosystems.

The midden surface and erosion layers were dominated by soil- and decomposition-associated taxa such as *Rahnella inusitata*, *Serratia fonticola*, *Raoultella terrigena*, *Pseudomonas yamanorum*, and *Sphingorhabdus soli* (Liu et al., 2020; Arnau et al., 2015), whereas the cliff and beach sediments contained a mixture of these lineages together with cold-adapted marine bacteria including *Marinobacter antarcticus*, *Shewanella frigidimarina*, and *Marinomonas primoryensis* (Liu et al., 2012; Bozal et al., 2002; Romanenko et al., 2003). By the tidal zone, the community was entirely marine, dominated by *Shewanella vesiculosa*, *Marinomonas polaris*, and *Pseudomonas viridilava*.

These observations indicate that while midden-associated bacteria can be detected within the eroding layers and nearby cliff sediments, but that the community composition changes rapidly along the transect and becomes dominated by marine taxa further downslope. Hence, microbes present in the midden appear to have very limited dispersal capacity once exposed, suggesting that erosion mainly results in local microbial release rather than long-range spread.

### 3.3 Resistome in Greenlandic middens

AMR (antimicrobial resistance) in microorganisms from environmental samples is often either intrinsic, i.e., innate to the organisms in their natural environment like soil, or extrinsic, which is related to animal-husbandry and other release of AMR-laden biological matter associated with, e.g., farming practices and sewage treatment plants. ARGs were present in all investigated sites, representing different ages of middens as well as infield and outfield soils. Across sites, 21 ARG classes were detected, with *β*-lactam and tetracycline resistance consistently among the most abundant (Supplementary Table S2). We also detected 647 distinct ARGs and per sample 2 to 101 ARGs were detected (median 10), with 2–3,610 ARG-assigned reads per sample.

At the Norse and early colonial era sites, ARGs affiliated with β-lactams, macrolides, lincosamides, and streptogramins were frequently observed (Supplementary Table S2). These classes include both intrinsic soil-associated resistance genes and determinants typical of faecal or livestock inputs, suggesting a mixed origin. Despite this diversity, overall ARG diversity did not differ between midden, infield, and outfield soils (Supplementary Table S2), indicating that the resistome is broadly distributed across the Norse farm landscape rather than restricted to the waste deposits.

At the Sermermiut Paleo-Inuit site, located adjacent to the sea, the resistome composition differed markedly between terrestrial cliff sediments and nearby marine foreland or beach sediments. The midden and cliff samples were dominated by *β*-lactam, macrolide, lincosamide, and aminoglycoside resistance genes, whereas marine samples exhibited higher ARG abundances and diversity (Supplementary Table S2). The elevated ARG abundance in marine sediments may reflect inputs from marine microbial communities naturally tolerant to antimicrobials, or more recent influence from human activities such as wastewater or human visitation in the area but not from the local midden. Several studies have shown a widespread occurrence of ARGs in pristine environments (Allen et al., 2008; D’costa et al., 2011) and it is challenging in metagenomic analyses to determine whether such genes are still located in their naive context or have been mobilized. In this study we used Resfinder which is a database only containing ARGs which have been found as mobilized and horizontally transferred. However, intrinsic variants will still be detected.

Overall, these data demonstrate that Greenlandic archaeological and natural soils host a wide array of resistance genes, most of which are consistent with long-term, intrinsic environmental reservoirs rather than modern contamination. However, the detection of clinically relevant classes such as *β*-lactam and macrolide resistance across both ancient and contemporary layers indicates that antimicrobial resistance determinants can persist over centuries. While no evidence suggests dissemination from middens into surrounding environments, ongoing permafrost thaw and erosion may gradually mobilize these ARGs, warranting continued monitoring as Arctic landscapes warm.

## 4 Conclusion

Our study focused on exploring ancient Arctic settlements in Greenland as potential reservoirs of bacterial pathogens through metagenome DNA sequencing. However, our findings should be interpreted as preserved microbial DNA signatures in archaeological deposits rather than authenticated ancient microbiomes or evidence of microbial viability.

Fortunately, we did not identify any potentially high-risk pathogenic strains. However, our findings revealed a distinct bacterial community composition within the studied Paleo-Inuit, Norse and early colonial era middens, contrasting significantly with the surrounding pristine soils. These middens contained a variety of human and animal commensal bacteria, alongside potential pathogens such as *C. perfringens* and *P. sordelii*, which were not found in nearby soils. The distribution of these commensal potentially pathogenic bacteria seems to be patchy and with the highest prevalence observed in the youngest midden (early colonial era) and in association with archeological artefacts. Notably, our study suggests limited potential for transport of bacteria from middens into the surrounding environment. Nonetheless, it underscores the importance of understanding and monitoring these potential pathogen sources to effectively mitigate and prevent outbreaks of infectious diseases.

## Data Availability

The datasets presented in this study can be found in online repositories. The names of the repository/repositories and accession number(s) can be found in the article/Supplementary material.

## References

[ref1] Abbott B. W. Jones J. B. (2015). Permafrost collapse alters soil carbon stocks, respiration, CH_4_, and N_2_O in upland tundra. Glob. Chang. Biol. 21, 4570–4587. doi: 10.1111/gcb.13069, 26301544

[ref2] Alempic J. M. Lartigue A. Goncharov A. E. Grosse G. Strauss J. Tikhonov A. N. . (2023). An update on eukaryotic viruses revived from ancient permafrost. Viruses 15:564. doi: 10.3390/v15020564, 36851778 PMC9958942

[ref3] Allen H. K. Moe L. A. Rodbumrer J. Gaarder A. Handelsman J. (2008). Functional metagenomics reveals diverse β-lactamases in a remote Alaskan soil. ISME J. 2009 3, 243–251. doi: 10.1038/ismej.2008.86 18843302

[ref4] Arnau V. G. Sánchez L. A. Delgado O. D. (2015). *Pseudomonas yamanorum* sp. nov., a psychrotolerant bacterium isolated from a subantarctic environment. Int. J. Syst. Evol. Microbiol. 65, 424–431. doi: 10.1099/ijs.0.065201-0, 25385990

[ref5] Aronoff D. M. (2013). *Clostridium novyi*, sordellii, and tetani: mechanisms of disease. Anaerobe 24, 98–101. doi: 10.1016/j.anaerobe.2013.08.009, 24036420

[ref6] Bartlett A. Padfield D. Lear L. Bendall R. Vos M. (2022). A comprehensive list of bacterial pathogens infecting humans. Microbiology (Reading). 168:001268. doi: 10.1099/mic.0.001269, 36748702

[ref7] Biagi E. Mengucci C. Barone M. Picone G. Lucchi A. Celi P. . (2020). Effects of vitamin B2 supplementation in broilers microbiota and metabolome. Microorganisms 8:1134. doi: 10.3390/microorganisms8081134 32727134 PMC7464963

[ref8] Biskaborn B. K. Smith S. L. Noetzli J. Matthes H. Vieira G. Streletskiy D. A. . (2019). Permafrost is warming at a global scale. Nat. Commun. 10:264. doi: 10.1038/s41467-018-08240-4, 30651568 PMC6335433

[ref9] Blanco-Míguez A. Beghini F. Cumbo F. Mciver L. J. Thompson K. N. Zolfo M. . Extending and improving metagenomic taxonomic profiling with uncharacterized species using MetaPhlAn 4. Nat. Biotechnol. 41, 1633–1644. doi: 10.1038/s41587-023-01688-w, 36823356 PMC10635831

[ref10] Bortolaia V. Kaas R. S. Ruppe E. Roberts M. C. Schwarz S. Cattoir V. . (2020). ResFinder 4.0 for predictions of phenotypes from genotypes. J. Antimicrob. Chemother. 75, 3491–3500. doi: 10.1093/jac/dkaa345, 32780112 PMC7662176

[ref11] Bozal N. Montes M. J. Tudela E. Jiménez F. Guinea J. (2002). Shewanella frigidimarina and *Shewanella livingstonensis* sp. nov. isolated from Antarctic coastal areas. Int. J. Syst. Evol. Microbiol. 52, 195–205. doi: 10.1099/00207713-52-1-195, 11837303

[ref12] Chen S. Zhou Y. Chen Y. Gu J. (2018). fastp: an ultra-fast all-in-one FASTQ preprocessor. Bioinformatics 34, i884–i890. doi: 10.1093/bioinformatics/bty560, 30423086 PMC6129281

[ref13] Clairbaux M. Fort J. Mathewson P. Porter W. Strøm H. Grémillet D. (2019). Climate change could overturn bird migration: transarctic flights and high-latitude residency in a sea ice free Arctic. Sci. Rep. 9:17767. doi: 10.1038/s41598-019-54228-5, 31780706 PMC6883031

[ref14] Clausen P. T. L. C. Hallgren M. B. Overballe-Petersen S. Marcelino V. R. Hasman H. Aarestrup F. M. (2025). Assembly-free typing of nanopore and Illumina data through proximity scoring with KMA. NAR Genom Bioinform. 7:lqaf116. doi: 10.1093/nargab/lqaf116, 40918069 PMC12408904

[ref15] D’costa V. M. King C. E. Kalan L. Morar M. Sung W. W. L. Schwarz C. . (2011). Antibiotic resistance is ancient. Nature 477, 457–461. doi: 10.1038/nature10388 21881561

[ref16] Dione N. Lo C. I. Raoult D. Fenollar F. Fournier P. E. (2020). *Clostridium massiliamazoniense* sp. nov., new bacterial species isolated from stool sample of a volunteer Brazilian. Curr. Microbiol. 77, 2008–2015. doi: 10.1007/s00284-020-02099-9, 32613254 PMC7415036

[ref17] Elberling B. Matthiesen H. Jørgensen C. J. Hansen U. Grønnow B. Meldgaard M. . (2011). Paleo-Eskimo kitchen midden Preservation in permafrost under future climate conditions at Qajaa, West Greenland. J. Archaeol. Sci. 38, 1331–1339. doi: 10.1016/j.jas.2011.01.011

[ref18] Ezhova E. Orlov D. Suhonen E. Kaverin D. Mahura A. Gennadinik V. . (2021). Climatic factors influencing the Anthrax outbreak of 2016 in Siberia, Russia. EcoHealth 18, 217–228. doi: 10.1007/s10393-021-01549-5, 34453636 PMC8463397

[ref19] Fenger-Nielsen R. Elberling B. Kroon A. Westergaard-Nielsen A. Matthiesen H. Harmsen H. . (2020). Arctic archaeological sites threatened by climate change: a regional multi-threat assessment of sites in south-West Greenland. Archaeometry 62, 1081–1297. doi: 10.1111/arcm.12593

[ref20] García S. Vidal J. E. Heredia N. Juneja V. K. (2023). “*Clostridium perfringens* infection,” in Food Microbiology: Fundamentals and Frontiers, (Hoboken, NJ: Wiley).

[ref21] Garrity G. M. Bell J. A. Lilburn T. G. (2004). Taxonomic Outline of the Prokaryotes Bergey’s Manual® of Systematic Bacteriology. Berlin: Springer.

[ref22] Günther A. Krone O. Svansson V. Pohlmann A. King J. Hallgrimsson G. T. . (2022). Iceland as stepping stone for spread of highly pathogenic avian influenza virus between Europe and North America. Emerg. Infect. Dis. 28, 2383–2388. doi: 10.3201/eid2812.221086, 36261139 PMC9707596

[ref24] Kembel S. W. Cowan P. D. Helmus M. R. Cornwell W. K. Morlon H. Ackerly D. D. . (2010). Picante: R tools for integrating phylogenies and ecology. Bioinformatics 26, 1463–1464. doi: 10.1093/bioinformatics/btq166, 20395285

[ref25] Koven C. D. Ringeval B. Friedlingstein P. Ciais P. Cadule P. Khvorostyanov D. . (2011). Permafrost carbon-climate feedbacks accelerate global warming. Proc. Natl. Acad. Sci. USA 108, 14769–14774. doi: 10.1073/pnas.1103910108, 21852573 PMC3169129

[ref26] Liu C. Chen C. X. Zhang X. Y. Yu Y. Liu A. Li G. W. . (2012). *Marinobacter antarcticus* sp. nov., a halotolerant bacterium isolated from Antarctic intertidal sandy sediment. Int. J. Syst. Evol. Microbiol. 62, 1838–1844. doi: 10.1099/ijs.0.035774-0, 21984673

[ref27] Liu Y. Du J. Zhang J. Lai Q. Shao Z. Zhu H. (2020). *Sphingorhabdus soli* sp. nov., isolated from Arctic soil. Int. J. Syst. Evol. Microbiol. 70, 1610–1616. doi: 10.1099/ijsem.0.003943, 31904318

[ref28] Madsen C. K. (2014). Pastoral Settlement, Farming, and Hierarchy in Norse Vatnahverfi, South Greenland. Copenhagen: University of Copenhagen.

[ref29] McMurdie P. J. Holmes S. (2013). Phyloseq: an R package for reproducible interactive analysis and graphics of microbiome census data. PLoS One 8:e61217. doi: 10.1371/journal.pone.0061217, 23630581 PMC3632530

[ref30] Neuwirth E. (2022). ColorBrewer Palettes [R Package RColorBrewer Version 1.1–3]. Vienna: R Foundation for Statistical Computing.

[ref31] Nielsen D. M. Pieper P. Barkhordarian A. Overduin P. Ilyina T. Brovkin V. . (2022). Increase in Arctic coastal erosion and its sensitivity to warming in the twenty-first century. Nat. Clim. Chang. 12, 263–270. doi: 10.1038/s41558-022-01281-0

[ref33] Oksanen J. Blanchet F. G. Friendly M. Kindt R. Legendre P. Mcglinn D. . (2019). Package “Vegan”, PR MinchinCommunity Ecology Package, Version, 2019. Vienna: R Foundation for Statistical Computing.

[ref34] Paradis E. Schliep K. (2019). Ape 5.0: an environment for modern phylogenetics and evolutionary analyses in R. Bioinformatics 35, 526–528. doi: 10.1093/bioinformatics/bty633, 30016406

[ref35] R Foundation for Statistical Computing (2023). A Grammar of Data Manipulation [R Package Dplyr Version 1.1.3]. Vienna: R Foundation for Statistical Computing.

[ref36] Rantanen M. Karpechko A. Lipponen A. Nordling K. Hyvärinen O. Ruosteenoja K. . (2022). The Arctic has warmed nearly four times faster than the globe since 1979. Commun. Earth Environ. 3:168. doi: 10.1038/s43247-022-00498-3

[ref37] Romanenko L. A. Uchino M. Mikhailov V. V. Zhukova N. V. Uchimura T. (2003). *Marinomonas primoryensis* sp. nov., a novel psychrophile isolated from coastal sea-ice in the sea of Japan. Int. J. Syst. Evol. Microbiol. 53, 829–832. doi: 10.1099/ijs.0.02280-0, 12807208

[ref38] Russel J. (2021). Russel88/MicEco: v0.9.15. Geneva: Zenodo.

[ref39] Russel J. Thorsen J. Brejnrod A. D. Bisgaard H. Sørensen S. J. Burmølle M. (2018). Datest: a framework for choosing differential abundance or expression method. bioRxiv:241802. doi: 10.1101/241802

[ref40] Slowikowski K (2026) ggrepel: automatically position non-overlapping text labels with ‘ggplot2’. R package version 0.9.8. Available online at: https://ggrepel.slowkow.com/authors#citation (Accessed November 6, 2023)

[ref42] Tréhard H. Poujol I. Mazuet C. Blanc Q. Gillet Y. Rossignol F. . (2016). A cluster of three cases of botulism due to *Clostridium baratii* type F, France, august 2015. Euro Surveill. 21, 1–5. doi: 10.2807/1560-7917.ES.2016.21.4.30117, 26848055

[ref43] Vonk J. E. Gustafsson Ö. (2013). Permafrost-carbon complexities. Nat. Geosci. 6, 675–676. doi: 10.1038/ngeo1937

[ref44] Westergaard-Nielsen A. Bjørnsson A. B. Jepsen M. R. Stendel M. Hansen B. U. Elberling B. (2015). Greenlandic sheep farming controlled by vegetation response today and at the end of the 21st century. Sci. Total Environ. 512-513, 672–681. doi: 10.1016/j.scitotenv.2015.01.039, 25679480

[ref45] Wickham H. (2016). ggplot2: Elegant Graphics for Data Analysis. New York, NY: Springer-Verlag.

[ref46] Wickham H. (2022). Simple, Consistent Wrappers for Common String Operations [R Package Stringr Version 1.5.0]. Vienna: R Foundation for Statistical Computing.

[ref47] Wickham H (2023) ggplot2 based publication ready plots ggpubr. Available online at: https://rpkgs.datanovia.com/ggpubr/ (Accessed November 6, 2023)

[ref48] Zastruzny S. F. Sjöberg Y. Jensen K. H. Liu Y. Elberling B. (2024). Impact of summer air temperature on water and solute transport on a permafrost-affected slope in West Greenland. Water Resour. Res. 60:e2023WR036147. doi: 10.1029/2023wr036147

