# Supplementary material for: Microbial composition of archaeological middens: tracing human footprints through centuries in Greenland’s ancient settlements
Source: Front Microbiol. 2026 Jun 17;17:1809037. doi: 10.3389/fmicb.2026.1809037 (PMC13319101; doi:10.3389/fmicb.2026.1809037)
Supplement: Supplementary file 1 [file Data_Sheet_1.DOCX]

SUPPLEMENTARY MATERIALS


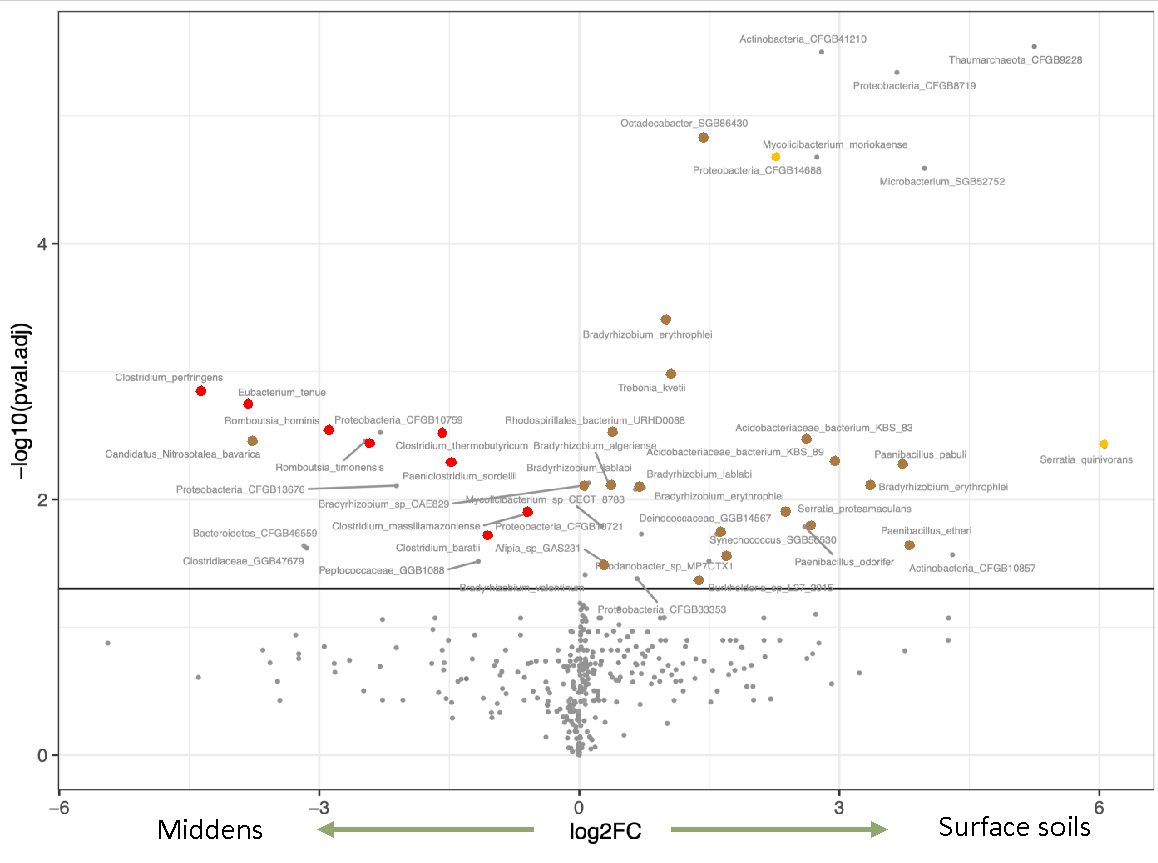


***Fig S1. Taxa enriched in middens versus soils****: volcano plot (fold change vs p-value) of differential abundance analysis (t-test) of bacterial taxa. Low-prevalence taxa were filtered, retaining only taxa present in at least five samples.Individual dots represent the taxa at species level, colored by their usual habitat: soil in brown, mixed in yellow, or host-associated in red, based on literature, and in grey if the taxonomic resolution is too low to identify species. Negative log2-fold change indicates increased relative abundance in middens while positive values indicate increased relative abundance in surface soils. Threshold p-value was set at < 0.05.*


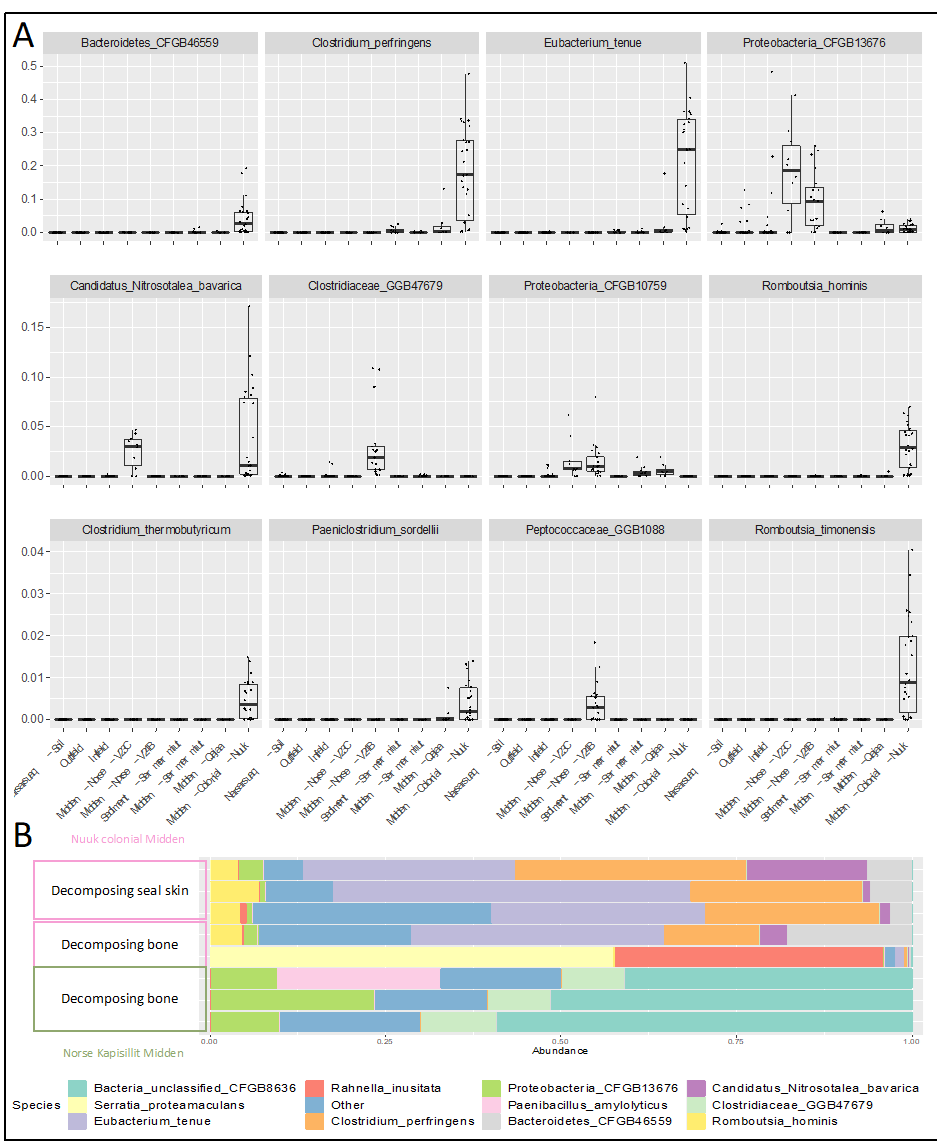


***FIG. S2. Midden-enriched taxa distribution****: A) Relative abundance of the species genomic bins enriched in middens compared to pristine soil across the sample types (middens: paleo-Inuit, Norse or colonial, and Norse infields and outfields, Narsarsuaq soil, Sermermiut sediment). B) Total microbial community composition of identified artifacts from Norse middens: Barplot of the 20 most abundant species (species with relative abundance lower than 2 % are grouped as ‘Other’).*
